# Deaths from Alzheimer’s Disease — United States, 1999–2014

**DOI:** 10.15585/mmwr.mm6620a1

**Published:** 2017-05-26

**Authors:** Christopher A. Taylor, Sujay F. Greenlund, Lisa C. McGuire, Hua Lu, Janet B. Croft

**Affiliations:** ^1^Division of Population Health, National Center for Chronic Disease Prevention and Health Promotion, CDC; ^2^Georgia State University, Atlanta, Georgia.

Alzheimer’s disease (Alzheimer’s), an ultimately fatal form of dementia, is the sixth leading cause of death in the United States, accounting for 3.6% of all deaths in 2014 (*1*,*2*). Alzheimer’s deaths can be an indicator of paid and unpaid caregiver burden because nearly everyone in the final stages of Alzheimer’s needs constant care, regardless of the setting, as the result of functional and cognitive declines (*2*). To examine deaths with Alzheimer’s as the underlying cause, state-level and county-level death certificate data from the National Vital Statistics System for the period 1999–2014 were analyzed. A total of 93,541 Alzheimer’s deaths occurred in the United States in 2014 at an age-adjusted (to the 2000 standard population) rate of 25.4 deaths per 100,000 population, a 54.5% increase compared with the 1999 rate of 16.5 deaths per 100,000. Most deaths occurred in a nursing home or long-term care facility. The percentage of Alzheimer’s decedents who died in a medical facility (e.g., hospital) declined from 14.7% in 1999 to 6.6% in 2014, whereas the percentage who died at home increased from 13.9% in 1999 to 24.9% in 2014. Significant increases in Alzheimer’s deaths coupled with an increase in the number of persons with Alzheimer’s dying at home have likely added to the burden on family members or other unpaid caregivers. Caregivers might benefit from interventions such as education, respite care, and case management that can lessen the potential burden of caregiving and can improve the care received by persons with Alzheimer’s.

Mortality data for 1999–2014 were analyzed using CDC WONDER (https://wonder.cdc.gov). The data were provided by the National Vital Statistics System and based on information from all resident death certificates filed in the 50 states and the District of Columbia (DC). The period analyzed represented all of the years with U.S. mortality data available at the time of analysis* using the *International Classification of Disease, Tenth Revision* (ICD-10) code set, which was implemented in 1999. CDC WONDER queries were used to generate the number of deaths with Alzheimer’s reported as the underlying cause of death, along with unadjusted and age-adjusted death rates with 95% confidence intervals and standard errors for groups defined by characteristics including year, sex, age group (≤64, 65–74, 75–84, and ≥85 years), race/ethnicity (non-Hispanic white, non-Hispanic black, American Indian/Alaska Native, Asian/Pacific Islander, or Hispanic), urban-rural classification, state, and county.

The percentages of Alzheimer’s deaths that occurred in medical facilities, the decedent’s home, hospice facility, or nursing home/long-term care facilities also were obtained. County-level data were examined for the aggregated years of 2005–2014 because the geographic distribution for 1999–2004 data were inconsistent with more recent data and would have obscured any current geographic patterns. ICD-10 codes G30.0, G30.1, G30.8, and G30.9 were used to identify Alzheimer’s as the underlying cause of death. These codes are used by CDC to describe Alzheimer’s as a leading cause of death (*1*). Other forms of dementia were not examined in this analysis.

Mortality rates were calculated using population estimates produced by the U.S. Census Bureau in collaboration with CDC’s National Center for Health Statistics. Age-adjusted mortality rates were calculated using the 2000 U.S. standard population. The z-statistic (assuming a normal approximation for the distribution of rates) was used to compare rates at a statistical significance level of p<0.05. No adjustment was made for multiple comparisons. Joinpoint regression was used to test the significance of trends in age-specific rates for the period 1999–2014.

From 1999 to 2014, age-specific rates of deaths attributed to Alzheimer’s increased among adults aged 75–84 years from 129.5 to 185.6 per 100,000 population and among adults aged ≥85 years, from 601.3 to 1,006.8. The largest increase in the rates of Alzheimer’s deaths among adults aged ≥85 years occurred from 1999 to 2005, compared with 2005–2014 (p<0.001) (Figure 1). Since 2005, although the mortality rate has continued to increase, the rate of increase was not as large as 1999–2005.

**FIGURE 1:**
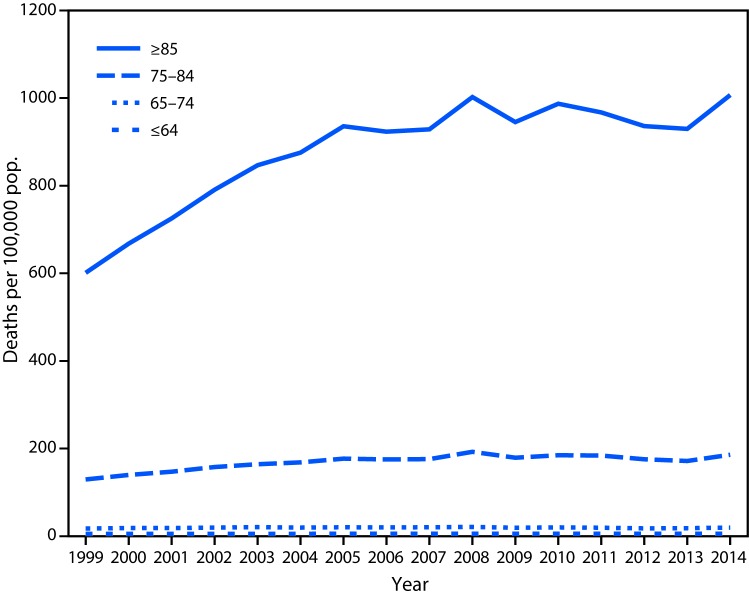
Death rates for Alzheimer’s disease as the underlying cause of death, by age group (years) — United States, 1999–2014

The age-adjusted Alzheimer’s death rate per 100,000 population increased from 16.5 (44,536 deaths) in 1999 to 25.4 (93,541 deaths) in 2014, an increase of 54.5% (Table). In 2014, rates were higher compared with 1999 among all age groups; also in 2014 rates were higher among women compared with men and among non-Hispanic whites compared with other racial/ethnic populations (Table). In 2014, death rates for Alzheimer’s were lower among residents of large central metropolitan areas and large fringe metropolitan areas compared with residents in other urban-rural classifications.

**TABLE:** Number, unadjusted rates, and age-adjusted rates per 100,000 population for Alzheimer’s disease deaths* as the underlying cause of death by age group, sex, race/ethnicity, urban-rural classification, and state — United States, 1999 and 2014

Characteristic	1999		2014		% change from 1999 to 2014
	No.	Rate (95% CI)	No.	Rate (95% CI)	
**Total**	**44,536**	**NA**	**93,541**	**NA**	**NA**
Unadjusted	NA	16.0 (15.8–16.1)	NA	29.3 (29.2–29.5)	83.8^†^
Age-adjusted**^§^**	NA	16.5 (16.3–16.6)	NA	25.4 (25.3–25.6)	54.5^†^
**Age group (yrs)**					
≤64	516	0.2 (0.2–0.2)	937	0.3 (0.3–0.4)	61.9^†^
65–74	3,204	17.4 (16.8–18.0)	5,170	19.6 (19.1–20.1)	12.5^†^
75–84	15,836	129.5 (127.5–131.6)	25,393	185.6 (183.3–187.9)	43.3^†^
≥85	24,980	601.3 (593.9–608.8)	62,041	1,006.8 (998.9–1,014.7)	67.4^†^
**Sex^§^**					
Male	13,391	14.4 (14.1–14.6)	28,362	20.6 (20.3–20.8)	43.1^†^
Female	31,145	17.4 (17.2–17.6)	65,179	28.3 (28.1–28.5)	62.7^†^
**Race/Ethnicity^§,^** ^¶^					
White, non-Hispanic	40,835	17.4 (17.3–17.6)	80,014	26.8 (26.6–27.0)	53.6^†^
Black, non-Hispanic	2,325	11.4 (10.9–11.9)	6,493	22.7 (22.2–23.3)	99.4^†^
American Indian/Alaska Native	86	10.4 (8.3–12.9)	287	18.7 (16.5–20.9)	80.1^†^
Asian/Pacific Islander	225	4.8 (4.2–5.5)	1,660	12.2 (11.6–12.7)	151.4^†^
Hispanic	981	9.6 (6.0–10.2)	4,934	19.8 (19.3–20.4)	107.2^†^
Urban-rural classification^§,^**					
Large central metro	11,582	15.3 (15.0–15.6)	23,964	23.7 (23.4–24.0)	55.0^†^
Large fringe metro	9,570	16.2 (15.8–16.5)	19,998	22.6 (22.3–22.9)	39.6^†^
Medium metro	9,776	17.5 (17.2–17.9)	22,083	28.0 (27.6–28.3)	59.6^†^
Small metro	4,816	18.1 (17.6–18.7)	10,160	27.9 (27.3–28.4)	53.7^†^
Micropolitan (nonmetro)	5,019	17.4 (16.9–17.9)	9,826	27.7 (27.2–28.3)	59.2^†^
Non-core (nonmetro rural)	3,773	15.5 (15.0–16.0)	7,510	27.1 (26.5–27.7)	74.9^†^
**State of residence^§,^** ^††^					
Alabama	772	17.8 (16.5–19.1)	1,885	35.3 (33.7–36.9)	98.3^†^
Alaska	24	11.9 (7.6–17.9)	68	17.2 (13.4–21.9)	44.5
Arizona	963	20.8 (19.5–22.1)	2,485	31.6 (30.3–32.8)	51.7^†^
Arkansas	434	14.8 (13.4–16.2)	1,193	34.8 (32.8–36.8)	134.5^†^
California	4,532	16.6 (16.1–17.1)	12,644	30.9 (30.4–31.5)	86.5^†^
Colorado	756	24.5 (22.7–26.2)	1,364	27.4 (25.9–28.9)	11.9^†^
Connecticut	449	11.4 (10.3–12.5)	923	18.4 (17.2–19.6)	61.6^†^
Delaware	107	15.0 (12.2–17.9)	188	16.6 (14.2–19.0)	10.5
District of Columbia	53	9.5 (7.1–12.4)	119	18.3 (15.0–21.7)	93.5^†^
Florida	3,059	14.3 (13.7–14.8)	5,874	18.8 (18.3–19.3)	31.8^†^
Georgia	1,080	18.8 (17.7–19.9)	2,670	31.7 (30.5–32.9)	68.9^†^
Hawaii	109	9.4 (7.7–11.2)	326	15.0 (13.4–16.7)	59.4^†^
Idaho	243	21.4 (18.7–24.1)	376	22.4 (20.1–24.7)	4.7
Illinois	1,908	15.9 (15.1–16.6)	3,266	21.9 (21.1–22.6)	38.0^†^
Indiana	1,106	18.9 (17.8–20.0)	2,204	29.4 (28.2–30.7)	55.7^†^
Iowa	706	18.2 (16.8–19.5)	1,313	29.6 (28.0–31.2)	62.8^†^
Kansas	511	16.6 (15.1–18.0)	790	21.9 (20.4–23.5)	32.3^†^
Kentucky	728	19.3 (17.9–20.7)	1,523	32.1 (30.4–33.7)	66.2^†^
Louisiana	683	17.9 (16.6–19.3)	1,670	36.0 (34.3–37.7)	101.1^†^
Maine	429	29.6 (26.8–32.4)	434	22.7 (20.5–24.8)	−23.5^†^
Maryland	681	15.4 (14.3–16.6)	934	14.5 (13.5–15.4)	−6.1
Massachusetts	1,182	16.5 (15.6–17.5)	1,688	19.0 (18.1–20.0)	15.3^†^
Michigan	1,431	15.4 (14.6–16.2)	3,349	27.0 (26.1–27.9)	75.2^†^
Minnesota	1,083	21.1 (19.8–22.4)	1,628	24.2 (23.0–25.4)	14.5^†^
Mississippi	356	13.3 (11.9–14.7)	1,098	35.2 (33.1–37.3)	164.1^†^
Missouri	914	15.0 (14.0–16.0)	2,053	27.4 (26.2–28.6)	82.9^†^
Montana	205	21.3 (18.4–24.3)	253	19.2 (16.9–21.6)	−9.9
Nebraska	331	16.3 (14.6–18.1)	515	21.9 (19.9–23.8)	33.8^†^
Nevada	174	13.6 (11.5–15.7)	606	23.8 (21.9–25.8)	75.2^†^
New Hampshire	266	23.2 (20.4–26.0)	396	24.0 (21.6–26.4)	3.5
New Jersey	1,041	12.0 (11.3–12.7)	1,962	17.4 (16.6–18.1)	44.8^†^
New Mexico	248	16.4 (14.4–18.5)	442	18.9 (17.1–20.7)	15.1
New York	1,357	7.0 (6.6–7.4)	2,639	10.7 (10.3–11.1)	52.2^†^
North Carolina	1,456	20.8 (19.7–21.9)	3,246	30.5 (29.5–31.6)	46.6^†^
North Dakota	155	18.1 (15.2–21.0)	364	36.2 (32.4–40.0)	99.7^†^
Ohio	2,099	18.2 (17.4–19.0)	4,083	27.7 (26.8–28.5)	51.8^†^
Oklahoma	553	15.4 (14.1–16.7)	1,227	28.9 (27.3–30.5)	87.5^†^
Oregon	866	24.1 (22.5–25.7)	1,411	28.5 (27.0–30.0)	17.9^†^
Pennsylvania	2,192	14.4 (13.8–15.0)	3,486	18.3 (17.7–18.9)	26.8^†^
Rhode Island	219	17.0 (14.7–19.2)	403	25.9 (23.3–28.6)	53.0^†^
South Carolina	690	20.5 (18.9–22.0)	1,938	37.4 (35.8–39.1)	83.0^†^
South Dakota	155	16.3 (13.7–18.9)	434	36.2 (32.7–39.6)	121.8^†^
Tennessee	944	17.9 (16.7–19.0)	2,672	38.1 (36.7–39.6)	113.1^†^
Texas	2,833	18.5 (17.8–19.2)	6,772	30.0 (29.3–30.7)	62.2^†^
Utah	245	17.3 (15.1–19.4)	584	26.7 (24.6–28.9)	54.8^†^
Vermont	127	20.5 (17.0–24.1)	266	31.9 (28.0–35.8)	55.2^†^
Virginia	917	15.9 (14.8–16.9)	1,775	20.8 (19.8–21.8)	31.2^†^
Washington	1,577	29.8 (28.3–31.2)	3,344	43.6 (42.1–45.1)	46.4^†^
West Virginia	314	15.0 (13.3–16.7)	620	25.5 (23.5–27.5)	69.7^†^
Wisconsin	1,170	19.9 (18.8–21.1)	1,876	25.0 (23.9–26.2)	25.5^†^
Wyoming	103	23.9 (19.3–28.5)	162	26.6 (22.5–30.8)	11.5

**Abbreviations:** CI = confidence interval; NA = not applicable.

* Alzheimer’s disease deaths in the National Vital Statistics System mortality file were identified using underlying cause-of-death *International Classification of Disease, Tenth Revision* codes G30.0, G30.1, G30.8, and G30.9.

^†^ Statistically significant difference (p<0.05) in rates for 1999 and 2014 using the z-statistic.

^§^ Age-adjusted death rates for all groups except age groups were standardized to the 2000 projected U.S. standard population.

**^¶^** Records without a specified Hispanic origin were excluded from this section.

** The National Center for Health Statistics urban-rural classification scheme classifies all U.S. counties into six levels that include large central metro (counties in metropolitan statistical areas [MSA] of ≥1 million population that also contain the entire population of the principal city of the MSA, or have their entire population contained in the largest principal city of the MSA, or contain at least 250,000 inhabitants of any principal city of the MSA); large fringe metro (counties in MSAs of ≥1 million population that did not qualify as large central metro counties; medium metro (counties in MSAs with populations of 250,000–999,999); small metro (counties in MSAs with populations <250,000); micropolitan (counties in a micropolitan statistical area that includes one or more urban clusters of 2,500–49,999 inhabitants that form the core and might contain outlying counties that meet specified requirements of commuting to or from the central counties); and noncore or rural nonmetropolitan counties that did not qualify as micropolitan.

^††^ State estimates are based on values from the entire state and not just from those counties that had available county-level data.

From 1999 to 2014, rates of Alzheimer’s deaths significantly increased for 41 states and DC (Table). Only one state, Maine, had a significant decrease in age-adjusted Alzheimer’s deaths. Age-adjusted rates for all 50 states and DC ranged from 7.0 to 29.8 per 100,000 in 1999 and from 10.7 to 43.6 per 100,000 in 2014.

Using average annual county-level data for the period 2005–2014, age-adjusted rates of Alzheimer’s deaths ranged from 4.3 to 123.7 per 100,000 (Figure 2). Counties with the highest age-adjusted rates were primarily in the Southeast, plus some additional areas in the Midwest and West.

**FIGURE 2:**
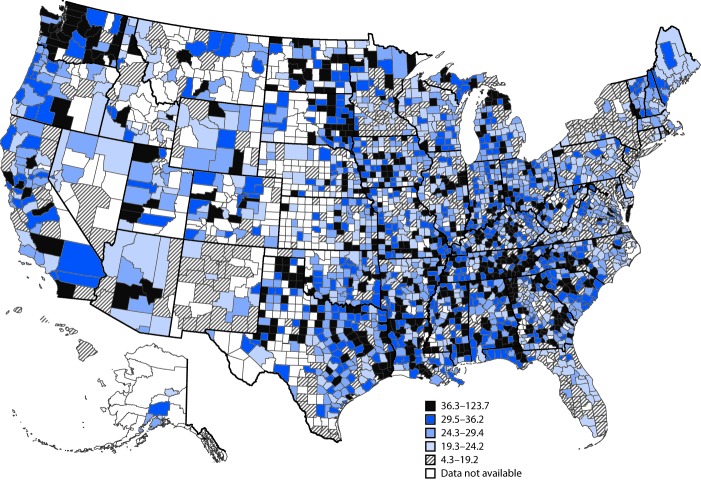
Average annual age-adjusted death rates from Alzheimer’s disease per 100,000 population, by county — United States, 2005–2014

Most Alzheimer’s decedents died in a nursing home or long-term care facility in 1999 (67.5%) and 2014 (54.1%). The percentage who died in a medical facility declined from 14.7% in 1999 to 6.6% in 2014. In contrast, the percentage who died at home increased from 13.9% in 1999 to 24.9% in 2014, with an additional 6.1% who died in a hospice facility in 2014.

## Discussion

Symptoms of early stage Alzheimer’s include memory loss that interferes with daily activities, difficulties with problem solving, losing or misplacing objects, and changes in mood and personality. As Alzheimer’s progresses, the brain’s ability to control language and reasoning becomes impaired. Persons might have problems recognizing family and friends or performing multistep tasks such as getting dressed. In advanced stages, persons with Alzheimer’s might be bedridden, have difficulty communicating, swallowing, or controlling bowel or bladder functions (*2*).

Adults aged ≥65 years are at greatest risk for developing Alzheimer’s (*2*). The number of Alzheimer’s deaths has increased, in part, because of a growing population of older adults. With the number of older adults increasing, the prevalence of Alzheimer’s is projected to quadruple by 2050 (*3*). However, age-adjusted rates of Alzheimer’s deaths have been increasing since 1979 (*4*). Although the actual number Alzheimer’s deaths might be increasing, the increase in the rate of Alzheimer’s deaths might also be attributed to increases in premorbid Alzheimer’s diagnosis by patients seeking care for symptoms and increased reporting by physicians, coroners, and medical examiners who assign causes of death.

Studies have shown that non-Hispanic blacks and Hispanics are more likely to have Alzheimer’s because of a wide variety of factors including increased cardiovascular disease risk factors (*5*). In contrast, this analysis showed that non-Hispanic whites have higher rates of Alzheimer’s deaths. The causes of the racial differences in the increase in Alzheimer’s death rates might be the result of competing causes of mortality; when compared with non-Hispanic whites, non-Hispanic blacks have higher rates for death from cardiovascular disease at younger ages (*6*).

It is important to note that the largest increase in the mortality rate occurred in older adults aged ≥85 years for the years 1999–2005. Since 2005, the mortality rate in this age group has continued to increase, but at a slower pace. This study did not directly examine factors that might have contributed to the sharp increase in reported deaths from 1999 to 2005 or the subsequent slowing of this increase. Increases in the mortality rate for Alzheimer’s might be the result of corresponding decreases in mortality rates for competing causes of death, including cardiovascular disease and stroke (*2*,*6*).

The increasing rates of Alzheimer’s deaths are not only problematic because of their obvious direct health effects on persons with Alzheimer’s. The debilitating nature of Alzheimer’s means that there are financial and societal costs borne by patients and their families, and by states and counties that operate publicly funded long-term care facilities. It is estimated that total health and long-term care costs for persons with Alzheimer’s and other dementias in the United States will total $259 billion in 2017, more than two thirds of which is expected to be covered by public sources such as Medicare and Medicaid (*2*). Additionally, most care provided to older adults with Alzheimer’s who do not live in long-term care facilities is provided by family members or other unpaid caregivers (*7*). In 2015, caregivers of persons with dementia, including Alzheimer’s, provided 18.2 billion hours of unpaid assistance (*2*). These caregiving hours might correspond to increased financial costs for caregivers and decreased work productivity, as caregivers might take leave from work to ensure adequate care is provided. The societal costs are substantial when considered in the context of the estimated 5.5 million U.S. residents who live with Alzheimer’s (*2*).

The findings in this report are subject to at least three limitations. First, several factors relating to the assigned cause of death might affect estimates of death involving Alzheimer’s. Evidence suggests that Alzheimer’s deaths reported on death certificates might be underestimates of the actual number of Alzheimer’s deaths in the United States (*8*). Because cases were identified using the underlying cause of death, persons with Alzheimer’s but a non-Alzheimer’s underlying cause of death were not identified in this analysis. Second, complications from Alzheimer’s, such as pneumonia, might be reported as the cause of death although the actual underlying cause of death, Alzheimer’s, was not reported on the death certificate. Finally, a person with Alzheimer’s might have dementia assigned as the underlying cause of death rather than a more specific diagnosis of Alzheimer’s.

Some modifiable risk factors for cardiovascular disease, such as obesity and fewer years of education, have been identified as factors associated with an increased risk for dementia (*9*,*10*). Although some treatments have been demonstrated to alleviate symptoms of Alzheimer’s, there is no cure or definitive means of prevention (*2*). Until Alzheimer’s can be prevented, slowed, or stopped, caregiving for persons with advanced Alzheimer’s will remain a demanding task. An increasing number of Alzheimer’s deaths coupled with an increasing number of patients dying at home suggests that there is an increasing number of caregivers of persons with Alzheimer’s. It is likely that these caregivers might benefit from interventions such as education, respite care, and case management that can lessen the potential burden of caregiving.

**Summary**

**What is already known about this topic?**

Alzheimer’s disease (Alzheimer’s) is the most common cause of dementia. It currently affects an estimated 5.5 million adults in the United States and is expected to affect 13.8 million U. S. adults aged ≥65 years by 2050.

**What is added by this report?**

Age-adjusted rates of Alzheimer’s mortality significantly increased in 41 states and the District of Columbia from 1999 to 2014. Counties with the highest age-adjusted rates were primarily in the Southeast, plus some additional areas in the Midwest and West. Significant increases in Alzheimer’s deaths coupled with an increase in the number of persons with Alzheimer’s dying at home suggest that the burden on caregivers has increased even more than the increase in the number of deaths.

**What are the implications for public health practice?**

Given the increasing number of Alzheimer’s deaths and persons with Alzheimer’s dying at home, there is a growing number of caregivers who likely can benefit from interventions like education, respite care, and home health assistance; such interventions can lessen the burden of caregiving and can improve the care received by persons with Alzheimer’s.

## Footnotes

* Before the release of 2015 National Vital Statistics System data on December 9, 2016.
